# Kids’ Environment and Health Cohort

**DOI:** 10.23889/ijpds.v9i5.1650

**Published:** 2024-09-18

**Authors:** Tolu Adedire, Esther Lewis, Dilly Stephenson, Nathan O'Connor, Leah Quinn, Hannah Goode

**Affiliations:** 1 Office for National Statistics; 2 Administrative Data Research UK (ADR UK)

This presentation discusses Kids’ Environment and Health Cohort (KEHC), a bespoke Administrative Data Research UK (ADR UK) funded linkage project. KEHC will be a comprehensive and yearly updated dataset for children born in England from 2006 onwards, integrating health, education, pregnancy, and environmental data. This integrates data from different administrative systems including the Office for National Statistics, the National Health Service, and the Department for Education as well as open environmental data.

This presentation will outline the deterministic and probabilistic linkage methods utilised and the quality assurance undertaken through clerical review and bias analysis. Additionally, we will delve into the challenges encountered in the process of data ingest, linkage, and delivery due to the diverse origins and purposes of the collected data, as well as temporal disparities. In navigating the intricacies of multiple linkages, we will discuss our unique methods, including plans to use an indexing first approach.

The first cohort of approximately 10 million children has been created to form the spine and further linkages to the 2011 and 2021 UK census data have been concluded. Linkages between address histories and open environmental data is ongoing. Linkage results have been promising, with high match rate and excellent quality.

KEHC is a powerful example of how integrated data can be used for research for the public good.

KEHC will provide a unique opportunity for academic researchers investigating the longitudinal effects of local environments on children’s health and educational outcomes and will become one of ADR UK’s flagship Research Ready Datasets.

